# Supplementary material for: Neprilysin Inhibition in the Prevention of Anthracycline-Induced Cardiotoxicity
Source: Cancers (Basel). 2023 Jan 3;15(1):312. doi: 10.3390/cancers15010312 (PMC9818213; doi:10.3390/cancers15010312)
Supplement: Supplementary file 1 [file cancers-15-00312-s001.zip › Table S1.pdf]

Table S1. ARNi administration in preclinical models of MI.

Paper by	Animal model	Gavage protocol	Drug dosage (mg/kg/d)*	LVEF (%)	Other findings*
Chang [1]	4-7-mo-old ♂ and ♀ Sprague-Dawley rats	Started 1 wk after MI (LVEF≤40%), cont. for 4 wks	Vehicle<br>Enalapril 20<br>ARNi 68	38.5 ± 2.0<br>46.7 ± 9.1 ↑<br>57.6 ± 5.5 ↑↑	↓↓ Heart weight/body weight ratio in ARNi<br>↓↓ Ventricular arrhythmias inducibility in ARNi<br>↑↑ Expression of K^+ channel proteins in ARNi
Chang [2]	♂ New Zealand White rabbits (2.5-4 kg)	Started 1 wk after MI (LVEF<40%), cont. for 4 wks	Vehicle<br>ARB 30<br>ARNi 60	37.1 ± 6.3<br>44.3 ± 6.3 ↑<br>53.8 ± 10.0 ↑↑	↓↓ Ventricular arrhythmia inducibility in ARNi
Ishii [3]	10-12-wk-old mice	Started 1 day after MI (FS < 30%), cont. for 4 wks	Vehicle<br>Enalapril 4<br>ARNi 20	NA	↓↓ Post-MI mortality rate due to LV rupture in ARNi<br>↑↑ FS 14 and 28 days post-MI in ARNi<br>↓↓ Myocardial expression of IL1β, IL6, and MMP-9 mRNA in ARNi<br>No differences in myocardial fibrosis and inflammatory infiltration between groups.
Kompa [4]	♂ Sprague-Dawley rats (250–300 g)	Started 1 wk after MI, cont. for 4 wks	Vehicle<br>Perindopril 2<br>ARNi 60	40.46 ± 1.27<br>42.22 ± 1.16<br>46.65 ± 0.83 ↑↑	ARNi significantly improved end-systolic pressure-volume relationship compared to perindopril<br>↓ LV mass, cardiomyocyte CSA, and cardiac fibrosis in perindopril and ARNi<br>↓↓ ANP, MHCβ, and TIMP2 gene expression in ARNi
Liu [5]	♂ 4-8-wk-old C57BL/6J mice	Started immediately after MI, cont. for 4 wks	Vehicle<br>Benazepril 10<br>ARNi 60<br>ARNi 60 + Benazepril 10	 =<br>58.7 ± 0.42 ↑<br>62.35 ± 0.25 ↑↑	Heart weight/body weight ratio ↓ in ARNi and benazepril and ↓↓ in ARNi + benazepril<br>Myocardial fibrosis ↓ in ARNi and ↓↓ in ARNi + benazepril<br>↓↓ TGFβ1 expression in ARNi and ARNi + benazepril<br>No differences in IL6 and TNFα expression between groups.
Pfau [6]	8-10-wk-old ♂ Lewis rats	Started 1 wk after MI	 Vehicle<br>ARB 31<br>ARNi 68	1 wk: 5 wks:<br>34 ± 2 35 ± 2<br>38 ± 2 39 ± 2<br>**39 ± 2** ↑ 42 ± 2	↓ Heart weight/tibial length ratio and fibrosis in ARNi<br>↓ Myocardial fibrosis in ARNi<br>↓ Myocyte CSA in ARNi and ARB<br>↓ Expression of CTGF, MHCβ, MHCβ/α, and ANP in ARNi and ARB
Raj [7]	♂ Sprague-Dawley rats (175–215 g)	Started immediately after MI, cont. for 8 wks	Vehicle<br>ARB 31<br>ARNi 68	56.60 ± 1.70<br>65.45 ± 2.70 ↑<br>66.82 ± 1.43 ↑	↓ Oxidative stress in ARNi and ARB<br>↓ TNFα, collagen, and BNP in ARNi and ARB
Shen [8]	♂ 3-mo-old Sprague-Dawley rats (260–300 g)	Started 1 wk after MI, cont. for 1 wk	Vehicle<br>ARNi 68	3 days: 7 days:<br><br>↑ ↑	↓ Interstitial fibrosis in ARNi<br>↓ Serum IL1βa and IL18 levels in ARNi<br>↓ ROS accumulation and NLRP3 inflammasome activation in ARNi
Suematsu [9]	8-10-wk-old ♂ C57BL/6J diabetic mice	Started day after MI, cont. for 4 wks	Vehicle<br>ARB 30<br>ARNi 60	29 ± 3.2<br>=<br>43 ± 3.4 ↑	↓↓ LV fibrosis and expression of TGFβ mRNA in ARNi<br>↓ Heart weight/body weight ratio in ARB and ARNi<br>↓ ANP mRNA in ARNi

Torrado [10]	♂ New Zealand White rabbits	I: at reperfusion only	Vehicle<br>ARB 9.1<br>ARNi 20	ARB =<br>ARNi ↑	↓ Infarct size in ARB and ARNi and ↓ cardiac troponin I serum concentration in ARNi
		II: started at LVEF ≤ 40%, cont. up to 10 wks		ARB =<br>ARNi ↑↑	↓ Infarct size in ARNi
		III: started at reperfusion, cont. for 10 wks		4 wks / 10 wks<br>ARB = / =<br>ARNi ↑↑ / ↑	
Trivedi [11]	♂ SHRs	Started 4 wks after reperfusion, cont. for 12 wks	Vehicle<br>ARB 31<br>ARNi 68	=<br>=<br>↑↑	↓ Infarct border zone expansion in ARB and ARNi<br>Aortic vasorelaxation responses to Ach and SNP ↑ in ARB and ↑↑ in ARNi<br>↑↑ Myocardial NO bioavailability in ARNi<br>No differences in fibrosis between groups
Vaskova [12]	6-8-wk-old ♀ Sprague-Dawley rats	Started 1 wk after MI, cont. for 7 wks	Vehicle<br>ARB 31<br>ARNi 68	36.79 ± 2.1<br>40.68 ± 4.8 ↑<br>41.42 ± 3.4 ↑	↑ Production of plasma exosomes in ARB and ARNi<br>↓↓ Expression of rno-miR-181a in ARNi<br>↓ Fibrosis in ARB and ARNi
von Lueder [13]	8-10-wk-old ♂ Lewis rats	Started 1 wk after MI, cont. for 4 wks	Vehicle<br>ARNi 68	47±5<br>60±2 ↑	Improved LV function in pressure-volume loops in ARNi<br>↓ LV mass and fibrosis in peri-infarct and remote myocardium in ARNi<br>No differences in infarct size and perivascular fibrosis between groups.

Abbreviations: Ach – acetylcholine; ANP – atrial natriuretic peptide; ARNi – sacubitril/valsartan, ARB – valsartan; cont. – continued; CSA – cross-sectional area; CTGF – connective tissue growth factor; FS – fractional shortening; IL – interleukin; LV – left ventricle; LVEF – left ventricle ejection fraction; MHCβ – myosin heavy chain β; MI – myocardial infarction; MMP – matrix metalloproteinase; mo – month; NA – not assessed; NLRP3 – NLR family pyrin domain containing 3; NO – nitric oxide; ROS – reactive oxygen species; SHRs – spontaneously hypertensive rats; SNP – sodium nitroprusside; TGFβ – transforming growth factor β; TIMP2 – tissue inhibitor of metalloproteinases 2; TNFα – tumor necrosis factor α; wk – week. Symbols: ↑/↓ – significantly increased/decreased compared to placebo; ↑↑/↓↓ – significantly increased/decreased compared to placebo and other groups; = – no significant change compared to placebo, ♂ – male, ♀ – female

* Groups and results presented in the Table S1 were chosen due to their importance for the review, they do not exhaust all of the results presented in selected papers.

References:

1. Chang, P.-C.; Lin, S.-F.; Chu, Y.; Wo, H.-T.; Lee, H.-L.; Huang, Y.-C.; Wen, M.-S.; Chou, C.-C. LCZ696 Therapy Reduces Ven-tricular Tachyarrhythmia Inducibility in a Myocardial Infarction-Induced Heart Failure Rat Model. Cardiovasc. Ther. 2019, 2019, 6032631.
2. Chang, P.-C.; Wo, H.-T.; Lee, H.-L.; Lin, S.-F.; Chu, Y.; Wen, M.-S.; Chou, C.-C. Sacubitril/Valsartan Therapy Ameliorates Ventricular Tachyarrhythmia Inducibility in a Rabbit Myocardial Infarction Model. J. Card. Fail. 2020, 26, 527–537.
3. Ishii, M.; Kaikita, K.; Sato, K.; Sueta, D.; Fujisue, K.; Arima, Y.; Oimatsu, Y.; Mitsuse, T.; Onoue, Y.; Araki, S.; et al. Cardio-protective Effects of LCZ696 (Sacubitril/Valsartan) After Experimental Acute Myocardial Infarction. JACC: Basic Transl. Sci. 2017, 2, 655–668.
4. Kompa, A.R.; Lu, J.; Weller, T.J.; Kelly, D.J.; Krum, H.; von Lueder, T.G.; Wang, B.H. Angiotensin receptor neprilysin inhibition provides superior cardioprotection compared to angiotensin converting enzyme inhibition after experimental myocardial infarction. Int. J. Cardiol. 2018, 258, 192–198.

5. Liu, Y.; Fan, Y.; Li, J.; Chen, M.; Chen, A.; Yang, D.; Guan, X.; Cao, Y. Combination of LCZ696 and ACEI further improves heart failure and myocardial fibrosis after acute myocardial infarction in mice. Biomed. Pharmacother. 2021, 133, 110824.
6. Pfau, D.; Thorn, S.L.; Zhang, J.; Mikush, N.; Renaud, J.M.; Klein, R.; Dekemp, R.A.; Wu, X.; Hu, X.; Sinusas, A.J.; et al. Angiotensin Receptor Neprilysin Inhibitor Attenuates Myocardial Remodeling and Improves Infarct Perfusion in Experimental Heart Failure. Sci. Rep. 2019, 9, 5791.
7. Raj, P.; Sayfee, K.; Parikh, M.; Yu, L.; Wigle, J.; Netticadan, T.; Zieroth, S. Comparative and Combinatorial Effects of Resveratrol and Sacubitril/Valsartan alongside Valsartan on Cardiac Remodeling and Dysfunction in MI-Induced Rats. Molecules 2021, 26, 5006.
8. Shen, J.; Fan, Z.; Sun, G.; Qi, G. Sacubitril/Valsartan (Lcz696) Reduces Myocardial Injury Following Myocardial Infarction by Inhibiting Nlrp3-Induced Pyroptosis Via the Tak1/Jnk Signaling Pathway. Mol. Med. Rep. 2021, 24, 676.
9. Suematsu, Y.; Miura, S.-I.; Goto, M.; Matsuo, Y.; Arimura, T.; Kuwano, T.; Imaizumi, S.; Iwata, A.; Yahiro, E.; Saku, K. LCZ696, an angiotensin receptor-neprilysin inhibitor, improves cardiac function with the attenuation of fibrosis in heart failure with reduced ejection fraction in streptozotocin-induced diabetic mice. Eur. J. Heart Fail. 2016, 18, 386–393.
10. Torrado, J.; Cain, C.; Mauro, A.G.; Romeo, F.; Ockaili, R.; Chau, V.Q.; Nestler, J.A.; Devarakonda, T.; Ghosh, S.; Das, A.; et al. Sacubitril/Valsartan Averts Adverse Post-Infarction Ventricular Remodeling and Preserves Systolic Function in Rabbits. J. Am. Coll. Cardiol. 2018, 72, 2342–2356.
11. Trivedi, R.K.; Polhemus, D.J.; Li, Z.; Yoo, D.; Koiwaya, H.; Scarborough, A.; Goodchild, T.T.; Lefer, D.J. Combined Angiotensin Receptor-Neprilysin Inhibitors Improve Cardiac and Vascular Function Via Increased No Bioavailability in Heart Failure. J. Am. Heart Assoc. 2018, 7, e008268.
12. Vaskova, E.; Ikeda, G.; Tada, Y.; Wahlquist, C.; Mercola, M.; Yang, P.C. Sacubitril/Valsartan Improves Cardiac Function and Decreases Myocardial Fibrosis Via Downregulation of Exosomal miR-181a in a Rodent Chronic Myocardial Infarction Model. J. Am. Heart Assoc. 2020, 9, e015640.
13. von Lueder, T.G.; Wang, B.H.; Kompa, A.R.; Huang, L.; Webb, R.; Jordaan, P.; Atar, D.; Krum, H. Angiotensin Receptor Neprilysin Inhibitor LCZ696 Attenuates Cardiac Remodeling and Dysfunction After Myocardial Infarction by Reducing Cardiac Fibrosis and Hypertrophy. Circ. Heart Fail. 2015, 8, 71–78.
