# Supplementary material for: Neprilysin Inhibition in the Prevention of Anthracycline-Induced Cardiotoxicity
Source: Cancers (Basel). 2023 Jan 3;15(1):312. doi: 10.3390/cancers15010312 (PMC9818213; doi:10.3390/cancers15010312)
Supplement: Supplementary file 1 [file cancers-15-00312-s001.zip › Table S2.pdf]

Table S2. ARNi administration compared to ARB or ACEi monotherapy in preclinical models of other cardiovascular diseases.

Model of	Paper by	Species	Gavage protocol	Drug dosage (mg/kg/d)*	Findings*
HFpEF due to pressure overload	Burke [1]	12-wk-old ♂ C57Bl6/J mice	Started a day before TAC, cont. for 4 wks	Vehicle<br>ARB 26<br>ARNi 57	↑↑ LVEF in ARNi<br>↓↓ Interstitial fibrosis and fibroblast population in ARNi<br>↓↓ Cardiomyocyte CSA and heart weight/tibial weight ratio in ARNi
	Lu [2]	♂ SD rats	Started 4 wks after TAC, cont. for 32 days	Vehicle<br>Enalapril 7<br>ARNi 30	↓↓ Sarcomere-length, LV fibrotic area, cardiomyocyte size and lung injury in ARNi<br>↓↓ Expressions of profibrotic, pro-oxidative, pro-apoptotic, DNA-damage, mitochondrial-damage and volume overload markers in LV in ARNi
	Norden [3]	♂ Sprague-Dawley rats	8 wks	Vehicle<br>ARB 31<br>ARNi 68	↓↓ LV weight in ARNi<br>↓↓ Diastolic dysfunction in ARNi<br>No differences in LVEF and myocardial fibrosis between groups
	Suo [4]	8-10-wk-old ♂ C57BL/6J mice	Started 8 wks after TAC, cont. for 4 wks	Vehicle<br>ARB 48<br>ARNi 60	↑↑ LVEF in ARNi<br>↓↓ Fibrosis in ARNi
HT	Hamano [5]	10-wk-old ♂ SHRcp fed high-salt diet	I: 6 mos, with high-salt diet<br>II: Started after 6 mos of high-salt diet, cont. for 6 mos	Vehicle<br>ARB 3<br>ARNi 6	↓ LV/body weight ratio in ARNi in Plan I<br>↓↓ LV/body weight and pulmonary edema in ARB in Plan II<br>No differences in cardiomyocyte CSA and fibrosis between groups
	Kusaka [6]	11-wk-old ♂ SHRcp fed high-salt diet	4 wks	Vehicle<br>ARB 30<br>ARNi 60	↓ LV in ARNi<br>↓↓ Myocardial fibrosis in ARNi<br>↓↓ Impairment of Ach-induced vascular relaxation in ARNi
	Seki [7]	8-11-mo-old SHRs	12 wks	Vehicle<br>ARB 20<br>ARNi 60	Endothelium-dependent hyperpolarization-mediated responses improved similarly in ARB and ARNi
	Sung [8]	18-wk-old ♀ SHRs	2 wks	Vehicle<br>ARB 160<br>ARNi 300	↓↓ Diastolic dysfunction and ↓↓ ventricular hypertrophy in ARNi<br>↓↓ Incidence of ventricular arrhythmias in ARNi<br>No differences in LVEF between groups
	Tashiro [9]	8-wk-old ♂ C57BL/6J mice	Started on the 7th day of Ang II infusion, cont. for 2 wks	Vehicle<br>Enalapril 12<br>ARB 30<br>ARNi 60	↓↓ LV concentric hypertrophy in ARNi<br>Myocyte CSA ↓ in ARB, ↓ in enalapril, and ↓↓ in ARNi<br>No differences in fibrosis and TGFβ expression between groups
	Zhao [10]	12-wk-old ♀ SHRs	12 wks	Vehicle<br>ARB 30<br>ARNi 60	LVEF ↑↑ in ARNi and ↑ in ARB<br>↓↓ LV mass in ARNi

					↓ Fibrosis, TGFβ expression and nNOS, eNOS protein expression in ARB and ARNi<br>↓↓ ACE, ATR1, and ↑↑ ACE2, MasR, ATR2 cardiac protein expression in ARNi
HF due to volume overload (by AVI)	Maslow [11]	♂ Sprague-Dawley rats	Started 4 wks after AVI, cont. for 4 wks.	Vehicle<br>ARB 31<br>Sac 31<br>ARNi 68	Improved load-dependent indexes of left ventricle contractility and relaxation only in ARNi<br>Improved load-independent index of contractility in ARB and ARNi<br>↑↑ Exercise tolerance in ARNi<br>↓↓ Myocardial fibrosis in ARNi
	Maslow [12]	♂ Sprague-Dawley rats	Started on the day of AVI, cont. for 8 wks.	Vehicle<br>ARB 31<br>NEPi 31<br>ARNi 68	↑ LVEF in ARNi<br>↓ Myocardial fibrosis in ARB, NEPi, and ARNi<br>↑ Exercise tolerance in ARB and ARNi
HFpEF due to obesity	Aroor [13]	Zucker Obese rats	Started at 16 wks of age, cont. for 10 wks	Vehicle<br>ARB 31<br>ARNi 68	↑ LVEF, ↓ fibrosis, and ↓ oxidative stress in ARB and ARNi<br>↑ Endothelial-dependent aortic relaxation in ARB and ARNi<br>↑↑ E'/a' ratio in ARNi
AF	Li [14]	♂ SPRD rats (200–250 g)	Started after AF induction, cont. for 4 wks	Vehicle<br>ARB 48<br>ARNi 60	↑ LVEF in ARB and ARNi<br>↓↓ Atrial fibrosis and susceptibility to AF in ARNi
Myocarditis	Liang [15]	6-wk-old ♂ BALB/c mice	Started on the day of myocarditis, cont. for 3 wks	Vehicle<br>ARB 10<br>ARNi 20	↓↓ Heart weight /body weight ratio, ↓↓ myocardial histopathologic scores, and ↓↓ cardiac troponin T levels in ARNi<br>↓↓ Serum hsCRP, IL6, and serum/myocardial IL17 levels in ARNi<br>↓↓ Th17 cells and their transcription factors in myocardial tissue in ARNi
CKD	Suematsu [16]	♂ SPRD rats (175–215 g)	Started 2 wks after nephrectomy, cont. for 8 wks.	Vehicle<br>ARB 30<br>ARNi 60	Heart weight/body weight ratio, myocyte CSA, markers of oxidative stress, myocardial and aortic fibrosis ↓↓ in ARNi and ↓ in ARB<br>↓↓ expression of NF-κB and COX-2 in ARNi

Abbreviations: ACE – angiotensin-converting enzyme; ACE2 – angiotensin-converting enzyme 2; Ach – acetylcholine; AF – atrial fibrosis; ARNi – sacubitril/valsartan; ARB – valsartan; ATR1 – angiotensin II receptor type 1; ATR2 – angiotensin II receptor type 2; AVI – aortic valve insufficiency; COX-2 – cyclooxygenase 2; CKD – chronic kidney disease; cont. – continued; CSA – cross-sectional area; eNOS – endothelial nitric oxide synthase; HFpEF – heart failure with preserved ejection fraction; hs CRP – high-sensitivity C-reactive protein; HT – hypertension; IL – interleukin; LV – left ventricle; LVEF – left ventricle ejection fraction; MasR – Mas receptor; mo – month; NEPi – sacubitril; NF-κB – nuclear factor kappa-light-chain-enhancer of activated B cells; nNOS – neuronal nitric oxide synthase; TAC – transverse aortic constriction; TGFβ – transforming growth factor β; SHRs – spontaneously hypertensive rats; wk - week. Symbols: ↑/↓ – significantly increased/decreased compared to placebo; ↑↑/↓↓ – significantly increased/decreased compared to placebo and other treatments, ♂ – male, ♀ – female.

* Groups and results presented in the Table were chosen due to their importance for the review, they do not exhaust all of the results presented in selected papers.

References

1. Burke, R.M.; Lighthouse, J.K.; Mickelsen, D.M.; Small, E.M. Sacubitril/Valsartan Decreases Cardiac Fibrosis in Left Ventricle Pressure Overload by Restoring PKG Signaling in Cardiac Fibroblasts. Circ. Heart Fail. 2019, 12, e005565..
2. Lu, H.-I.; Tong, M.-S.; Chen, K.-H.; Lee, F.-Y.; Chiang, J.Y.; Chung, S.-Y.; Sung, P.-H.; Yip, H.-K. Entresto therapy effectively protects heart and lung against transverse aortic constriction induced cardiopulmonary syndrome injury in rat.. Am. J. Transl. Res. 2018, 10, 2290–2305.
3. Nordén, E.S.; Bendiksen, B.A.; Andresen, H.; Bergo, K.K.; Espe, E.K.; Hasic, A.; Hauge-Iversen, I.M.; Veras, I.; Hussain, R.I.; Sjaastad, I.; et al. Sacubitril/valsartan ameliorates cardiac hypertrophy and preserves diastolic function in cardiac pressure overload. ESC Heart Fail. 2021, 8, 918–927.
4. Suo, Y.; Yuan, M.; Li, H.; Zhang, Y.; Li, Y.; Fu, H.; Han, F.; Ma, C.; Wang, Y.; Bao, Q.; et al. Sacubitril/Valsartan Improves Left Atrial and Left Atrial Appendage Function in Patients With Atrial Fibrillation and in Pressure Overload-Induced Mice. Front. Pharmacol. 2019, 10, 1285.
5. Hamano, G.; Yamamoto, K.; Takami, Y.; Takeshita, H.; Shimosato, T.; Moritani, T.; Rakugi, H. Effects of Low-Dose Sacubi-tril/Valsartan on Different Stages of Cardiac Hypertrophy in Salt-Loaded Hypertensive Rats. J. Cardiovasc. Pharmacol. 2019, 73, 282–289.
6. Kusaka, H.; Sueta, D.; Koibuchi, N.; Hasegawa, Y.; Nakagawa, T.; Lin, B.; Ogawa, H.; Kim-Mitsuyama, S. LCZ696, Angiotensin II Receptor-Neprilysin Inhibitor, Ameliorates High-Salt-Induced Hypertension and Cardiovascular Injury More Than Valsartan Alone. Am. J. Hypertens. 2015, 28, 1409–1417.
7. Seki, T.; Goto, K.; Kansui, Y.; Ohtsubo, T.; Matsumura, K.; Kitazono, T. Angiotensin II Receptor–Neprilysin Inhibitor Sacu-bitril/Valsartan Improves Endothelial Dysfunction in Spontaneously Hypertensive Rats. J. Am. Heart Assoc. 2017, 6, e006617.
8. Sung, Y.L.; Lin, T.T.; Syu, J.Y.; Hsu, H.J.; Lin, K.Y.; Liu, Y.B.; Lin, S.F. Reverse Electromechanical Modelling of Diastolic Dys-function in Spontaneous Hypertensive Rat after Sacubitril/Valsartan Therapy. ESC Heart Fail. 2020, 7, 4040–4050.
9. Tashiro, K.; Kuwano, T.; Ideishi, A.; Morita, H.; Idemoto, Y.; Goto, M.; Suematsu, Y.; Miura, S.I. Sacubitril/Valsartan Inhibits Cardiomyocyte Hypertrophy in Angiotensin Ii-Induced Hypertensive Mice Independent of a Blood Pressure-Lowering Effect. Cardiol. Res. 2020, 11, 376–385.
10. Zhao, Y.; Ma, R.; Yu, X.; Li, N.; Zhao, X.; Yu, J. AHU377+Valsartan (LCZ696) Modulates Renin–Angiotensin System (RAS) in the Cardiac of Female Spontaneously Hypertensive Rats Compared With Valsartan. J. Cardiovasc. Pharmacol. Ther. 2019, 24, 450–459.
11. Maslov, M.Y.; Foianini, S.; Mayer, D.; Orlov, M.V.; Lovich, M.A. Interaction Between Sacubitril and Valsartan in Preventing Heart Failure Induced by Aortic Valve Insufficiency in Rats. J. Card. Fail. 2019, 25, 921–931.
12. Maslov, M.Y.; Foianini, S.; Mayer, D.; Orlov, M.V.; Lovich, M.A. Synergy between sacubitril and valsartan leads to hemo-dynamic, antifibrotic, and exercise tolerance benefits in rats with preexisting heart failure. Am. J. Physiol. Circ. Physiol. 2019, 316, H289–H297.
13. Aroor, A.R.; Mummidi, S.; Lopez-Alvarenga, J.C.; Das, N.; Habibi, J.; Jia, G.; Lastra, G.; Chandrasekar, B.; DeMarco, V.G. Sacubitril/Valsartan Inhibits Obesity-Associated Diastolic Dysfunction through Suppression of Ventricular-Vascular Stiffness. Cardiovasc. Diabetol. 2021, 20, 80.
14. Li, S.N.; Zhang, J.R.; Zhou, L.; Xi, H.; Li, C.Y.; Zhao, L. Sacubitril/Valsartan Decreases Atrial Fibrillation Susceptibility by Inhibiting Angiotensin Ii-Induced Atrial Fibrosis through P-Smad2/3, P-Jnk, and P-P38 Signaling Pathways. J. Cardiovasc. Transl. Res. 2022, 15, 131–142.
15. Liang, W.; Xie, B.K.; Ding, P.W.; Wang, M.; Yuan, J.; Cheng, X.; Liao, Y.H.; Yu, M. Sacubitril/Valsartan Alleviates Experimental Autoimmune Myocarditis by Inhibiting Th17 Cell Differentiation Independently of the Nlrp3 Inflammasome Pathway. Front. Pharmacol. 2021, 12, 727838.
16. Suematsu, Y.; Jing, W.; Nunes, A.; Kashyap, M.L.; Khazaeli, M.; Vaziri, N.D.; Moradi, H. LCZ696 (Sacubitril/Valsartan), an Angiotensin-Receptor Neprilysin Inhibitor, Attenuates Cardiac Hypertrophy, Fibrosis, and Vasculopathy in a Rat Model of Chronic Kidney Disease. J. Card. Fail. 2018, 24, 266–275.
