# Supplementary material for: Neprilysin Inhibition in the Prevention of Anthracycline-Induced Cardiotoxicity
Source: Cancers (Basel). 2023 Jan 3;15(1):312. doi: 10.3390/cancers15010312 (PMC9818213; doi:10.3390/cancers15010312)
Supplement: Supplementary file 1 [file cancers-15-00312-s001.zip › Table S3.pdf]

Table S3. ARNi administration in animal models of anthracycline-induced cardiotoxicity.

**Paper by**	**Animal model**	**Group size**	**Dox protocol**	**Gavage protocol**	**Drugs + Dosage (mg/kg/d)**	**Other findings** (in comparison to DOX + Vehicle groups)
Boutagy [1]	11-12-wk-old ♂ Wistar rats	*n* = 25 each	2.15 mg/kg ip every 3rd day for 3 wks (up to 15 mg/kg)	Started on the day of Dox, cont. for 6 wks	Sham - no Dox<br>Dox + Vehicle<br>Dox + ARB 31<br>Dox + ARNi 68	↑ LVEF in ARNi<br>↓ Myocyte vacuolation in ARNi and ARB<br>↓ Myocardial fibrosis in ARNi and ARB at 4 wks (no longer seen at 6 wks)<br>↓ Capillary density in ARNi at 6 wks<br>↓ MMP activity in ARNi at 4 wks<br>↓ Myocyte CSA and heart weight in all Dox-receiving groups<br>No differences in cellular apoptosis between groups
Dindas [2]	7–8-wk-old ♂ Balb-c mice	*n* = 6 each	20 mg/kg ip. – 1 dose on 5th day of gavage	Started 4 days before Dox, cont. for 9 days	Vehicle<br>ARNi 80<br>Dox + Vehicle<br>Dox + ARNi 80	↓ Degenerative changes and streaking in cardiomyocytes in Dox + ARNi<br>↓ QRS duration, ST interval and QT/PQ index in Dox + ARNi<br>↓ NT-proBNP, TNF-α, IL-1β, IL-6, caspase 3 immunoreactivity in DOX + ARNi<br>↓ Total oxidant status and ↑ total antioxidant status in DOX + ARNi
Kim [3]	♂ Sprague-Dawley rats	*n* = 8 each	5 mg/kg ip. q1w for 3 wks (up to 15 mg/kg)	Started 1 day before Dox, cont. for 6 wks	Vehicle<br>Dox + Vehicle<br>Dox + ARNi 60	↓ Cardiomyocyte apoptosis in ARNi group<br>↓ Endoplasmic reticulum stress levels in ARNi group<br>↓ Serum troponin I and NT-proBNP levels in ARNi group
Miyoshi [4]	♂ Sprague-Dawley rats	n = 15 each	1.5 mg/kg ip. for 10 days (up to 15 mg/kg) followed by 8 days of no treatment	Started on the day of Dox, cont. for 18 days	Sham - no Dox<br>Dox + Vehicle<br>Dox + Val 31<br>Dox + Sac/Val 68	No differences in LVEF and FS<br>↑ Cardiomyocyte cross-sectional area in ARNi<br>↓ Cardiac fibrosis in ARNi<br>↓ Cardiac TNFα and ANP mRNA expression in ARB and ARNi<br>↓ Collagen I mRNA expression in ARNi<br>↓ Cardiac troponin T and NT-proBNP levels in ARNi<br>↓ Cardiac reactive oxygen species levels in ARNi<br>↑ Phosphorylation of AMPK and ↓ Bax/Bcl-2 ratio in ARNi
**Maurea [5]**	♀ C57Bl/6 mice	n = 6 each	2.17 mg/kg ip. daily for 10 days	Cont. for 10 days	Sham<br>Sac/Val 60<br>Dox<br>Dox + Sac/Val 60	ARNi improved EF and prevented the reduction of radial and longitudinal strain<br>↓ Cardiac expression of NLRP3, MyD88, DAMPs, and NF-kB in ARNi<br>↑ Expression of phosphorylated AMPK in ARNi<br>↓ Levels of Calgranulin S100 and galectine-3 in ARNi
Xia [6]	8-wk-old ♂ Balb-c mice	*n* = 7 each	15 mg/kg ip. 3x/wk for 2 wks	Started the day after Dox, cont. for 4	Vehicle<br>Dox + Vehicle	↓ Cardiac hypertrophy, myocardial fibrosis and cellular apoptosis in ARNi<br>↓ Heart weight/body weight ratio and ↑ heart weight/tibial length ratio in

				wks,	Dox + ARNi 80	ARNi<br>↑ Single cardiomyocyte contractile function in ARNi<br>↓ Pathologic morphologic changes to mitochondria and ↓ myocardial Drp1 expression in ARNi<br>↓ Cleaved caspase 3 level in ARNi
Ye [7]	8-wk-old ♂ C57BL/6 mice	n = 7 each	5 mg/kg ip. q1w for 3 wks (up to 15 mg/kg)	Started the day after Dox, cont. 6 wks,	Vehicle<br>Dox + Vehicle<br>Dox + ARNi 60<br>TLR2 KO + Vehicle<br>TLR2 KO + Dox	↑ LVEF in ARNi, TLR2 KO, and TLR2 KO + Dox<br>↓ Thinning of the ventricular wall and enlargement of the heart cavity in ARNi and TLR2 KO<br>↓ Myocardial fibrosis in ARNi and TLR2 KO<br>↓ Myocardial collagen I and TGF-b protein levels in ARNi and TLR2 KO<br>↓ Myocardial TNFα and NF-κB levels in ARNi and TLR2 KO
Yu [8]	12-wk-old ♂ New Zealand white rabbits	n = 5 each	1.5 mg/kg iv. q1w for 5 wks (up to 7.5 mg/kg)	Started one wk after Dox, cont. for 4 wks	Vehicle<br>Dox + Vehicle<br>Dox + ARNi 5<br>Dox + ARNi 10	↓ PR segment, QRC segment prolongation, QT interARB and QT/PQ index in ARNi groups<br>↓ Serum BNP level in ARNi groups<br>↑ Activity of superoxide dismutase and catalase and ↓ lipid peroxidation in ARNi groups

Abbreviations: AMPK – adenosine monophosphate-activated protein kinase; ANP – atrial natriuretic peptide; ARB – valsartan; ARNi – sacubitril/valsartan; BNP – brain natriuretic peptide; CSA – cross-sectional area; Dox – doxorubicin; Drp1 – dynamin-related protein 1; KO – knock-out; IL – interleukin; LVEF – left ventricle ejection fraction; NF-κB - nuclear factor kappa-light-chain-enhancer of activated B cells; NT-proBNP – N-terminal prohormone of brain natriuretic peptide; TGFβ – transforming growth factor β; TNFα – tumor necrosis factor α; q1w – every week; wk – week. Symbols: ↑ / ↓ – significantly increased/decreased, ♂ – male, ♀ – female.

References

1. Boutagy, N.E.; Feher, A.; Pfau, D.; Liu, Z.; Guerrera, N.M.; Freeburg, L.A.; Womack, S.J.; Hoenes, A.C.; Zeiss, C.; Young, L.H.; et al. Dual Angiotensin Receptor-Neprilysin Inhibition With Sacubitril/Valsartan Attenuates Systolic Dysfunction in Experi-mental Doxorubicin-Induced Cardiotoxicity. JACC Cardiooncology 2020, 2, 774–787.
2. Dindaş, F.; Güngör, H.; Ekici, M.; Akokay, P.; Erhan, F.; Doğduş, M.; Yılmaz, M.B. Angiotensin Receptor-Neprilysin Inhibition by Sacubitril/Valsartan Attenuates Doxorubicin-Induced Cardiotoxicity in a Pretreatment Mice Model by Interfering with Oxidative Stress, Inflammation, and Caspase 3 Apoptotic Pathway. Anatol. J. Cardiol. 2021, 25, 821–828.
3. Kim, B.S.; Park, I.-H.; Lee, A.-H.; Kim, H.-J.; Lim, Y.-H.; Shin, J.-H. Sacubitril/valsartan reduces endoplasmic reticulum stress in a rat model of doxorubicin-induced cardiotoxicity. Arch. Toxicol. 2022, 96, 1065–1074.
4. Miyoshi, T.; Nakamura, K.; Amioka, N.; Hatipoglu, O.F.; Yonezawa, T.; Saito, Y.; Yoshida, M.; Akagi, S.; Ito, H. LCZ696 ameliorates doxorubicin-induced cardiomyocyte toxicity in rats. Sci. Rep. 2022, 12, 4930.
5. Maurea, N.; Canale, M.L.; Buccolo, S.; Bisceglia, I.; Paccone, A.; Maurea, F.; Scherillo, M.; Quagliariello, V. Abstract 12402: Sacubitril-Valsartan Increases Pampk and Reduces Inflammasome, Myddosome, Il-6 and Galectin-3 Levels in Short-Term Doxorubicin-Treated Mice Improving Longitudinal Strain and Ejection Fraction. Circulation 2022, 146, A12402.

6. Xia, Y.; Chen, Z.; Chen, A.; Fu, M.; Dong, Z.; Hu, K.; Yang, X.; Zou, Y.; Sun, A.; Qian, J.; et al. LCZ696 improves cardiac function via alleviating Drp1-mediated mitochondrial dysfunction in mice with doxorubicin-induced dilated cardiomyopathy. J. Mol. Cell. Cardiol. 2017, 108, 138–148.
7. 48. Ye, S.; Su, L.; Shan, P.; Ye, B.; Wu, S.; Liang, G.; Huang, W. Lcz696 Attenuated Doxorubicin-Induced Chronic Cardiomyopathy through the Tlr2-Myd88 Complex Formation. Front. Cell Dev. Biol. 2021, 9, 654051.Yu, C.; Li, D.; Li, Z.; Yu, D.; Zhai, G. Effect of sacubitril/valsartan on inflammation and oxidative stress in doxorubicin-induced heart failure model in rabbits. Acta Pharm. 2021, 71, 473–484.
